# Feasibility, Safety and Effects of a One-Week, Ski-Based Exercise Intervention in Brain Tumor Patients and Their Relatives: A Pilot Study

**DOI:** 10.3390/jcm9041006

**Published:** 2020-04-02

**Authors:** Fabian M. Troschel, Christian Ramroth, Lars Lemcke, Jens Clasing, Amelie S. Troschel, Martin Dugas, Walter Stummer, Rainer Wiewrodt, Ralf Brandt, Dorothee Wiewrodt

**Affiliations:** 1 Department of Radiation Oncology, Münster University Hospital, 48149 Münster, Germany; 2 Department of Neurosurgery, Münster University Hospital, 48149 Münster, Germany; 3 Department of Surgery, Raphaelsklinik, 48143 Münster, Germany; 4 Institute of Medical Informatics, Münster University Hospital, 48149 Münster, Germany; 5 Pulmonary Division, Department of Medicine A, Münster University Hospital, 48149 Münster, Germany; 6 Personal Training Brandt, 48165 Münster, Germany

**Keywords:** glioma, glioblastoma multiforme, brain neoplasms, exercise, feasibility study, quality of life, rehabilitation, depression, psycho-oncology, caregivers

## Abstract

A brain tumor diagnosis poses a significant psychological burden and it severely impacts quality of life (QOL), both in patients and relatives. However, comprehensive strategies addressing QOL in this setting remain rare. Here, we aim to share our findings of a one-week ski exercise intervention, with emphasis on feasibility, safety, QOL, and physical exercise. The intervention consisted of week-long daily ski sessions with professional ski guides as well as dedicated physicians present. The participants were handed questionnaires, including distress and QOL items before, during, and after the intervention. Using fitness watches, exercise intensity was also tracked at these timepoints. During the intervention, patients were checked for adverse events daily. Fifteen participants, nine patients after multidisciplinary treatment, and six relatives were included in the study. Additionally, 13 children participated in the exercise, but not in the study. All of the participants completed the entire program. No severe adverse events were documented during daily checks. There was a strong increase in quantified activity and QOL with a corresponding decrease in distress during the intervention, and, partly, afterwards. This prospective brain tumor rehabilitation study demonstrates the feasibility and safety of challenging ski exercise in brain tumor patients. The findings also underline the exercise-mediated QOL benefits, emphasizing the need for more comprehensive brain tumor rehabilitation programs.

## 1. Introduction

Brain tumors, such as glioblastoma multiforme (GBM), pose both a high physical and psychological burden for patients given intensive therapy [1], disease-related symptoms [2], and, in many cases, unfavorable prognosis [3].

In recent years, quality of life (QOL) has increasingly come into focus as a key outcome parameter in brain tumor patients [4]. In this cohort, both anxiety disorders and depression may be present in more than 40% of patients, despite only 5% of patients having been diagnosed with a psychiatric illness before brain tumor diagnosis [5]. Close to three-quarters of primary brain tumor patients may experience distress over the course of their disease [6]. Surveys, such as the Hospital Anxiety and Depression Scale (HADS), indicate that, among all cancer patients, brain tumor patients may be especially vulnerable for anxiety and depression [7]. Importantly, relatives also experience helplessness, anxiety, and depression in their roles as caregivers [6]. Thus, QOL-based studies are needed in brain tumor patients to develop a comprehensive toolset of interventions to counteract distress [4,8].

Exercise has only rarely been offered to brain tumor patients due to caregivers’ fear of epileptic seizures and falls [9,10]. The German GBM guidelines explicitly discourage exercise in this patient cohort [11]. Only recently, some case series and case reports indicated that exercise is tolerable in brain tumor patients [12,13,14]. Our group reported a case of a GBM patient who participated in a two-year training program and completed two marathon runs under multimodal therapy with no adverse events [15]. However, many questions remain, and exercise therapy is still rare and insufficiently investigated in brain tumor patients.

Given the lingering uncertainty as well as lack of literature in the field, the present prospective pilot study explores another exercise program in this setting. We aim to share our experience regarding a ski-based exercise intervention in brain tumor patients and their relatives concerning feasibility and safety as well as effects on activity and QOL measures.

## 2. Materials and Methods

The ethics committee of the Westfalian Wilhelms-University and the Medical Association Westphalia-Lippe approved this prospective cohort study (No. 2019-027-f-S). All of the participants consented to the study. The study protocol adhered to the principles that were set forth in the Helsinki declaration.

### 2.1. Inclusion

**Figure 1:**
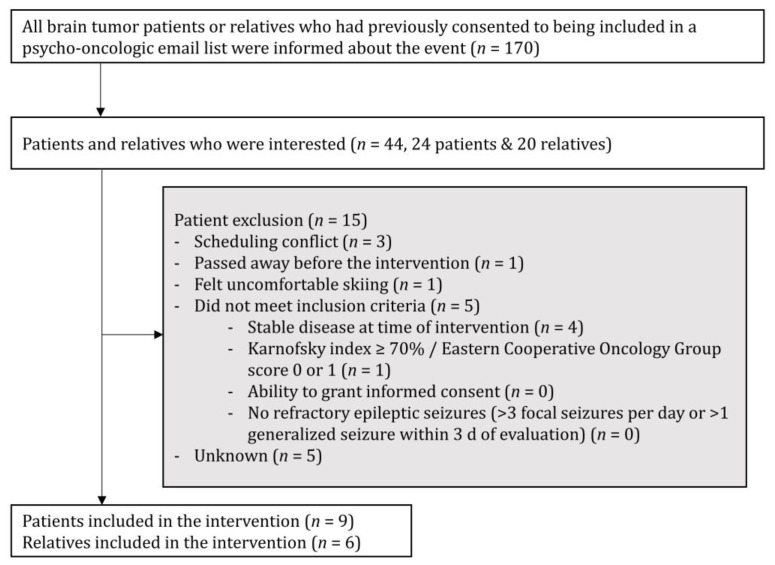
Consort diagram with inclusion and exclusion criteria.

In a first step, for recruitment, we tapped into a psycho-oncologic email list containing email addresses from patients and relatives (Figure 1). All of the recipients had previously consented to their inclusion on the list that was used to promote both theoretical psycho-educational content as well as offers for arts and exercise workshops for psycho-oncologic treatment. Patients were asked to informally indicate interest in participating.

In a second step, more detailed plans were shared with those interested and the exact dates were given. The inclusion criteria were then applied to the interested patients. Finally, the patients were given the opportunity to make a final decision concerning participation in November 2018. Cost of the entire intervention was covered by the institutional support fund (please see acknowledgements). If affordable for them, patients were asked, but not required, to contribute to the Münster University Hospital Central Nervous System Association (Förderverein Zentrales Nervensystem e.V.).

### 2.2. Intervention

In February 2019, a pre-event meeting was held to allow participants to get to know each other. The main intervention took place between 9 and 16 March 2019, as a full-week ski-based exercise intervention. Patients and family met at the university hospital on the first day and they were transferred via bus to the ski lodge. The lodge was located adjacent to a ski slope and was connected to the valley via ski lift. It was set at 1400 m above sea level and the surroundings were constantly snow-covered (at least three feet) during the week, necessitating care navigating and requiring additional strength and balance while outside.

The cabin was entirely booked by the study group and other groups did not use it during this time. Given it was a self-catering lodge, food was prepared by a cook as part of the team with the help of participants and evenings were spent together as a group. At night, the participants stayed in shared rooms with their families. During the day, there was a 2h-ski session in the morning and in the afternoon, weather permitting. The group was subdivided according to prior ski experience and preference (alpine skiing or cross-country skiing). Thus, the group sizes varied between two and five participants. Three board certified physicians (one trauma surgeon, two neurosurgeons, one of them with additional board certifications in psychotherapy and psychooncology) were present at all times. Daily, in the afternoon, the patients were checked for serious adverse events (SAE) and non-serious adverse events (NSAE), as detailed in Table S1.

### 2.3. Fitness Tracker and QOL Questionnaires

During the pre-meeting, each adult participant received a fitness watch (Polar M430, Polar Electro, Kempele, Finland), free of charge. The patients were given a short introduction to the watch and were then asked to constantly wear it in the week preceding the intervention, during the intervention and post-intervention. For analysis, four consecutive days of the week prior to intervention, during intervention, and of the week post intervention were compared in terms of hours defined as active by the training watch and in terms of calories burnt during these days. For inclusion, the participants had to wear the watch at least 15 h a day on average (at least 60 h total), allowing for the removal of the watch during the night.

During the pre-meeting, mid-way through the intervention week, one month, and two months post-intervention, the participants received an electronic questionnaire on a smartphone. The app (for Google Android and Apple iOS) was developed based on the “my patient data” platform (http://meine-patientendaten.de/) with this app, data are locally stored and transferred into the study database via a two-dimensional (2D) barcode. The following items were included:

- From the European Organization for Research and Treatment of Cancer (EORTC) Core Quality of Life Questionnaire (QLQ) C30 questionnaire, self-reported health and self-reported quality of life were obtained (both on a scale from 1 to 7, from worst to best). These items were first used in patients with non-resectable lung cancer, but they are recommended for all cancer patients [16].
- The World Health Organization Well-Being Index (WHO-5; in % from worst (0) to best (100)) is a commonly used questionnaire for well-being in over 30 languages all over the world. It includes five positively-phrased statements regarding quality of life in the two weeks prior to testing with a scale of five options, graded from 0 to 5 each [17]. The raw score, which was obtained by building the sum of all five values, ranges from 0 to 25, and is then multiplied by 4 for a range from 0 to 100.
- The three Allgemeine Selbstwirksamkeit Kurzskala (ASKU; Köln, German, translates to Short General Self-Efficacy Index) items (all on a scale from 1 to 5 from worst to best) were included to monitor the changes in self-efficacy expectations [18]. The scale was developed in German for any adult German-speaking population. However, it is also available in English [18].
- The HADS questionnaire (including seven items each for anxiety (HADS-A) and depression (HADS-D), scaled from 0 to 3 individually from best to worst and then summed for scores from 0 to 21) is a well-known scale to grade both anxiety and depression and it has found ample use in oncologic settings [7].
- The distress thermometer is a simple one-item scale that requires patients to grade their individual level of distress from 0 to 10, from least to most distress [19].
- Finally, the participants were asked to indicate any physical problems (present/not present) from a list of 20 items, which was based on the distress thermometer problem list [20].

**Figure 2:**
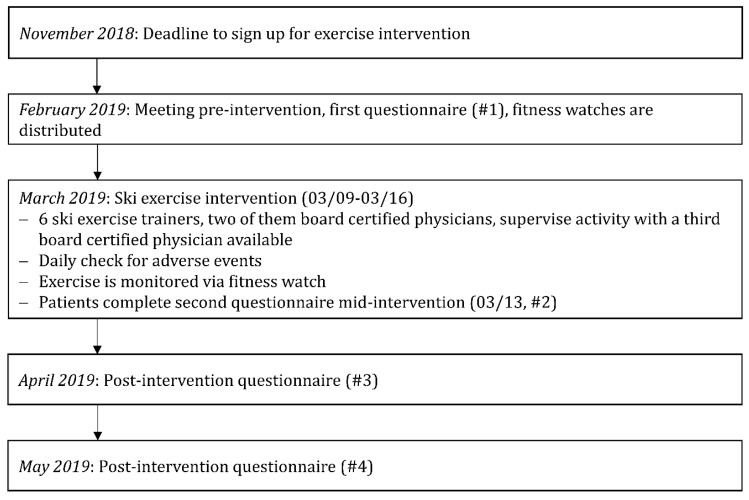
Sequence of events for this study, visualized using a flow chart.

Figure 2 summarizes the sequence of events.

### 2.4. Statistics

GraphPad Prism (GraphPad Software, Inc, San Diego, CA, USA) was used for data visualization. Given low numbers of participants, we refrained from evaluating differences for statistical significances. The patient characteristics were analyzed using respective median and range. Prevalence data were summarized as absolute and relative frequencies. Fitness data was given as mean relative to the pre-intervention week. The questionnaire data were given as mean on the respective item scale. Not all of the participants answered all the questionnaire items. Given the low numbers, no attempt was made to impute missing values. Instead, the patients were dropped from individual item analyses if they had not consistently completed respective items across all four timepoints.

## 3. Results

### 3.1. Recruitment

The email list that we used during the first step of recruitment consisted of 170 email addresses at the time of its use for the exercise intervention. Forty-four recipients (24 patients, 20 relatives) indicated interest after this first presentation. During the further planning process, 15 patients dropped out: In most cases, the reason was either “scheduling conflict” or “other unknown reason”, accounting for a total of eight cases. In one case, the patient felt uncomfortable skiing. One patient passed away before the intervention. Finally, five patients were excluded, as they did not meet the inclusion criteria, given acute disease progression (*n* = 4) and the Karnofsky index < 70% (*n* = 1, Figure 1).

### 3.2. Participants

A total of 15 participants were included, nine patients and six relatives. With them, 10 children also took part. While the children also received skiing lessons, they were not subject to this study. Table 1 lists the patient characteristics. Patient diagnoses included four high-grade and four low-grade gliomas as well as one case of multilocular meningioma. Most of the patients presented with symptoms at diagnosis and they all had undergone prior resections. Seven patients had also received cranial radiotherapy with doses of 50 to 60 Gy in all cases as well as chemotherapy, most commonly with temozolomide. Five patients had been diagnosed with a recurrence of their disease at least once over the course of their treatment up to the intervention. Of note, three patients received therapy during the exercise intervention: two were under temozolomide and one received tumor treating field (TTF) therapy.

Besides, 10 team members participated: there were six ski instructors, including the personal training expert, who conducts the institution’s brain tumor physical exercise program since 2011 (R.B.) and two doctors (one neurosurgeon and one trauma surgeon). The principal investigator who is both a neurosurgeon and a psycho-oncologist, one member of the study staff, as well as a cook and a housekeeper were the other participants.

### 3.3. Adverse Events

No serious adverse events were noted during daily checks. One patient described an increase in numbness in his right hand that had started before the intervention and persisted throughout. His daily cortisone dose was escalated during the intervention and he still participated in the entire program. Follow-up imaging revealed tumor growth of his GBM most likely responsible for the symptom.

### 3.4. Fitness Data

All but one patient and all relatives met inclusion criteria, wearing the watch at least 60 h over each four-day interval. On sum, the participants wore the watch for similar total times across all three timepoints (Figure S1), thus allowing for direct comparison between the three intervals.

**Figure 3:**
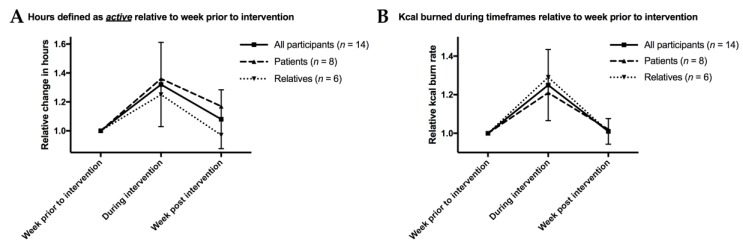
Fitness data as quantified by a fitness watch. Inclusion criteria for this analysis included wearing the fitness watch at least 60 h over a four-day period the week before, during and after the intervention. The data is presented relative to pre-intervention levels. (**A**): Hours defined as active by the fitness watch during respective timeframes. (**B**): Kcal burned during respective timeframes. For all figures, the mean results are given for all participants as well as separately for patients and relatives. To demonstrate variability, standard deviation bars are given for “all participants”.

During the intervention, the time of physical activity as quantified by the training watch increased by more than one third (Figure 3A), with a slightly stronger increase among patients when compared to relatives. The week after intervention, the patients continued to be approximately 10% more active on average when compared to before the intervention, while there was no strong difference among relatives. Kilocalorie burn rate, meanwhile, was also increased during the exercise intervention (Figure 3B). However, post-intervention, there were no meaningful changes to before the exercise.

### 3.5. Quality of Life Data

**Figure 4:**
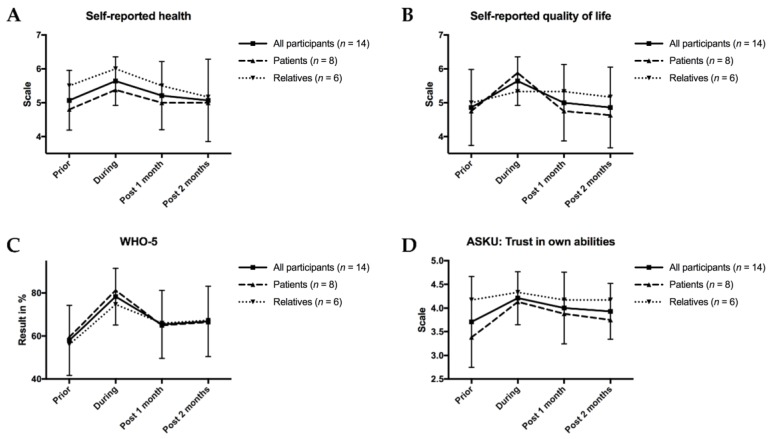
Quality of life measures according to participants’ questionnaires. (**A**,**B**): Self-reported health and quality of life on a scale from 1 to 7 from worst to best. (**C**)World Health Organization-5 (WHO-5) scale from 0% (worst) to 100% (best). (**D**): Allgemeine Selbstwirksamkeit Kurzskala (ASKU) item trust in own abilities, from 1 (worst) to 5 (best). For all figures, mean results are given for all participants as well as separately for patients and relatives. To demonstrate variability, standard deviation bars are given for “all participants”.

The patients were asked to complete several well-established questionnaire items over four timepoints. Interestingly, all of the QOL items spiked during the intervention (Figure 4): Self-reported health and self-reported QOL were highest during the intervention (Figure 4A–B). The relatives reported higher self-reported health than patients. Well-being, as quantified by the WHO-5 index, increased by roughly 20% compared to before the intervention (Figure 4C). Notably, trust in own abilities as determined by the ASKU score was higher among patients during and at both times after the intervention when compared to before (Figure 4D). Meanwhile, the other two ASKU items showed no strong differences before, during, or after the intervention (Figure S2).

**Figure 5:**
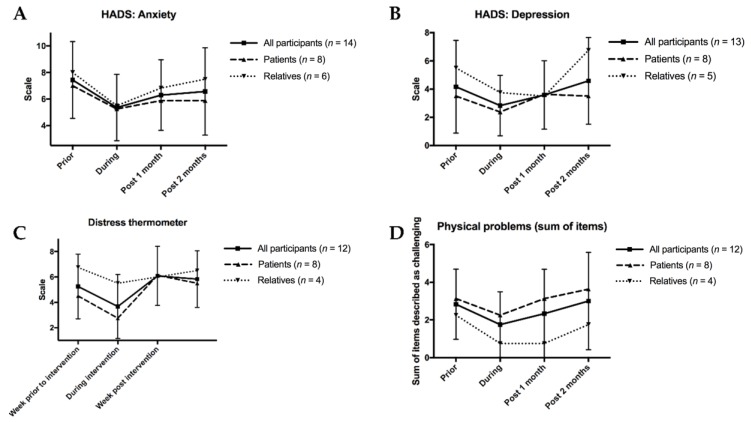
Anxiety, depression, distress and physical problems according to participants’ questionnaires. (**A**): Anxiety, as measured by the Hospital Anxiety and Depression Scale (HADS) questionnaire (HADS-A) scale as a measure of anxiety, ranging from 0 (best) to 21 (worst). (**B**): Depression, as measured by the HADS questionnaire (HADS-D) scale as a measure of depression, ranging from 0 (best) to 21 (worst). (**C**): Distress, as measured by the distress thermometer, ranging von 0 (least) to 10 (most). (**D**): Physical problems as a sum of 20 items with patients indicating if they found each physical task challenging (1) or not challenging (0), with the scale thus ranging from 0 (best) to 20 (worst). For all figures, mean results are given for all participants as well as separately for patients and relatives. To demonstrate variability, standard deviation bars are given for all participants.

Another set of questionnaire items centered on anxiety, depression and distress (Figure 5): Anxiety, as measured by the HADS questionnaire (HADS-A), was the lowest among all groups during the intervention and remained comparatively low at the following two timepoints (Figure 5A). Depression, which was the second HADS item (HADS-D), was also the lowest during the intervention in patients, although no long-term changes were visible (Figure 5B). The distress thermometer corroborated these findings, as it indicated the lowest distress levels during the intervention (Figure 5C). Consistently across most timepoints, all three items showed relatives, not patients, describing more anxiety, depression, and distress, respectively. Lastly, the sum of physical challenges was also the lowest during the intervention (Figure 5D). We then compared the relation between psychological and physical symptom in patients and relatives. For this, we summed all of the continuously available HADS-A, HADS-D, and distress thermometer scores and then divided them by the physical problem score (Figure S3). Patients’ comparatively low ratio between distress, depression and anxiety, and physical symptoms indicated that they suffered from both problem fields. However, in relatives, psychological burden far surpassed physical challenges.

## 4. Discussion

The purpose of this study was threefold: We wanted to share our experience with this ski-based experience intervention in brain tumor patients with regard to feasibility and safety, effects on activity, and QOL.

### 4.1. Feasibility, Safety and Adverse Events

In this study, we demonstrate that a ski-based exercise intervention is feasible in pre-selected brain tumor patients. There were no SAEs and all patients completed the entire, intensive one-week program. Despite fear of epileptic seizures sometimes found in the literature [9,10], none occurred.

Thus, this study further undercuts the general discouragement that still persists in some guidelines [11] regarding exercise in brain tumor patients. In fact, there is an increasing number of studies supporting that exercise might be feasible in brain tumor patients: A 12-week exercise program was implemented in 24 brain tumor patients, with 14 completing the entire program and no adverse events were noted [12]. A six-week program was attended by a single case, who subsequently improved in balance, walking, and QOL, again with no adverse events [14]. Another 12-week program in two cases was again well-tolerated and improved QOL [13]. In a case reported by our group, a two-year exercise program, including two marathon runs, did not produce any adverse effects [15]. Based on questionnaires, a significant number of patients continue to exercise at home during and after brain tumor treatment long-term on their own [21]. Finally, a newly-published systematic review in patients suffering from epilepsy summarizes there is no basis to generally discourage exercise in this patient cohort [22].

To the best of our knowledge, this study is the first to implement ski-based exercise in brain tumor patients. Winter sports may be met with significant skepticism in this population, given the challenging and complex nature of the exercise, with adequate balance playing a key role in preventing accidents [23]. Additionally, navigating in the snow—even without skis—requires more balance and is more exhausting. However, even winter sports are possible in a well-tailored setting with pre-selected patients, which makes even more questionable the general discouragement from any sport that is given to some brain tumor patients, as our study demonstrates.

### 4.2. Patient Selection

Instead of general discouragement, for exercise interventions, such as this one, we recommend detailed patient selection. Our study patients experienced substantial therapy—all of them had previously undergone a tumor resection, most of them received cranial irradiation and/or chemotherapy, and three were under ongoing therapy. Patients mainly carried challenging diagnoses, including GBM and other high-grade gliomas. Thus, the participants were representative of the brain tumor population. Nonetheless, we clearly defined exclusion criteria for patients who experienced tumor progression, a high frequency of epileptic seizures, and who may not have been able to adequately tolerate exercise due to severe physical impairment. To this end, in the past and present, including patients with a Karnofsky Index of ≥70%, has worked well in other studies as well as in our prior investigations. We believe our inclusion criteria allow for the inclusion of a significant segment of brain tumor patients, but readily acknowledge that not all brain tumor patients meet our inclusion criteria and that this intervention is not suitable for all patients. In the times of personalized medicine for molecular cancer treatment [24], rehabilitation and exercise should also consider the individual patient’s case and ability.

### 4.3. Effects on Activity and Future Implications

During the course of their disease, patients commonly experience increasing frailty as a loss of muscle function [25]. Meanwhile, 45% of brain tumor patients would like to receive information regarding participating in an exercise program, while 47% felt able to participate in such a program even during treatment [21]. From a medical perspective, offering exercise in brain tumor patients makes sense, too, given effects on physical decline, cognitive impairment and emotional status, and, potentially, survival [26,27]. Thus, there is a strong rationale to offer exercise to brain tumor patients.

However, studies show that, among brain tumor patients, muscular strength is only 60% and cardiorespiratory fitness only 40% of predicted values subsequent to surgery [28] with the 6-min. walk test also impaired [29], raising serious doubts about the practicability of high-intensity exercise in this population.

In our study, even challenging ski-based exercise proved feasible: we used fitness watches to quantify patients’ exercise and demonstrate that, during the intervention, activity and kilocalorie burn rate were both improved by roughly 30% and 25%, respectively. This shows that brain tumor patients are able to tolerate a significant exercise increase in a short time period and underlines that even intensive exercise regimens may be implemented. A one-week ski-based exercise is known to improve balance in recreational skiers [30], a key challenge in brain tumor patients [26]. Given that, in prostate cancer, exercise was even able to reverse loss of muscle [31], with muscle being key to survival in several cancer entities [32,33,34], the implementation of challenging exercise might even have implications for survival.

### 4.4. Effects on Quality of Life

Given the effects of both the tumor and its therapy on physical, emotional, and cognitive abilities, maintaining QOL is a key concern among brain tumor patients [26,35]. Among others, the effects on everyday life include cognitive impairment [36], loss of autonomy and coping with bad prognosis [37], all leading to a significant decline in QOL [35,37]. Brain tumor patients are especially vulnerable regarding anxiety and depression, according to the HADS score [7].

However, exercise might have the potential to counteract QOL decline and it might also improve QOL-related physical impairments [26]. This is also reflected in our study, as we quantified several QOL items longitudinally:

- Self-reported health and QOL, as measured by the EORTC-QLQ-C30 items [16], were both improved during the intervention. It should be noted that patients connected with each other in the setting of the intervention, as all participants (and the study team) stayed in the same lodge that they had for themselves. Friendships formed and they have led to multiple meeting among patients and their relatives since. Moreover, the program allowed for patients and their families to get out of their daily routine and enjoy the intervention days without the setbacks of everyday life. While these aspects were not directly quantified by our items, the participants spoke highly of the intervention’s social spirit.
- The WHO-5 questionnaire, a well-validated and widely-used well-being measure [17], also demonstrated strong improvements in patients and relatives, further underlining the positive effects the intervention had on QOL.
- Interestingly, the ASKU questionnaire, an item that measures subjective competence expectations [18], showed strong improvements among patients during the exercise with minimal changes among relatives. It is well-conceivable that patients gained more self-trust during their experience skiing. Based on the longitudinal quantification, this trust seems to have lasted, even after the intervention, which indicated that patients reacted well to the challenging setting of winter sports. The other two ASKU items were not directly connected to the exercise and, thus, showed little effects.

Conversely, anxiety, depression, and distress all decreased during the intervention:

- Before the intervention, HADS-A and HADS-D were both largely in line with a previous study in brain tumor patients [38]. Both indicators decreased during the intervention with a stronger effect in the anxiety item. Meeting the challenge of alpine skiing might have contributed to a growth in self-trust and, subsequently, a decline in anxiety. Interestingly, the lower anxiety persisted longitudinally.
- While the Distress thermometer, a well-established tool [19], showed some decreases, especially among patients, during the intervention, no long-term effects were seen. In fact, distress was somewhat increased post intervention when compared to before the intervention. This was most likely due to the fact that two of the patients faced progression of their disease at the time of the first post-intervention questionnaire. This underlines that it is challenging to adequately evaluate the long-term changes of the intervention.
- Interestingly, all three items showed relatives indicating more symptoms and distress than patients on average. This is consistent with prior studies highlighting the needs of caregivers in the family: at brain tumor diagnosis, 38% of patients and 78% of caregivers exhibit strong distress [39]. On average, the distress thermometer measurements are two points higher in caregivers when compared to patients at diagnosis and at recurrence [39]. This matches perfectly with the pre-intervention distress thermometer results of our study. By comparing psychological and physical challenges, we show that, while patients suffer from problems that stem from both fields, there is a large psychological burden in physically healthy relatives. Our study contributes to a growing body of literature indicating a strong need to also focus on relatives, as they experience high distress and anxiety levels [40,41].
- To the best of our knowledge, this study is the first in the brain tumor field to investigate family caregivers along with patients in an exercise setting. We show that family caregivers also need QOL interventions and profit from interventions like ours.

It remains important to stress that the organization of this kind of intervention remains challenging and we recommend the consideration of the following potential hurdles.

Some patients may feel skeptical about skiing (or other sports) given their condition and might thus be less likely to participate. This point was sometimes raised in our recruitment. To this end, this pilot study might provide an encouragement for patients, as we demonstrate that neither the altitude nor the kind and intensity of the exercise precluded brain tumor patients from successfully participating in the program.

This kind of exercise intervention is costly, which limits its appeal. It is true that significant resources, both in terms of finances and personnel, were needed for this study. However, this investigation might also serve to encourage smaller, less expensive exercise interventions, as it demonstrates the feasibility of exercise in brain tumor patients.

It is important to keep in mind that not all brain tumor patients may be suitable for this kind of intervention. We do not claim that exercise may benefit all patients—in fact, the primary goal of our study is to demonstrate that careful pre-selection among brain tumor patients might yield a patient population that is suitable for exercise, as discussed above. This would already be a significant departure from general discouragement of exercise in brain tumor patient, as is currently widely practiced.

There are some important limitations to this study:

First, and most importantly, the number of participants was low, and the small sample size requires caution when interpreting the study results given the chance of false-positive or false-negative results. While we decided not to employ detailed statistical analysis for this reason, this also remains a limitation for the trends that we show. However, this study was designed as a pilot study, given that, to our knowledge, ski-based exercise has never been attempted in brain tumor patients. Additionally, exercise interventions are difficult to organize on a large scale, especially in brain tumor patients, which is reflected by the fact that, despite the relatively small sample size, this is one of the larger studies in a field that is dominated by case reports.

Second, we only assessed the participants’ activity, but not their fitness. Similar to other cancer entities, like lung cancer, evaluating fitness would necessitate further tests [42] and/or imaging [34].

Third, it is vital to keep in mind that, besides only exercise, other aspects may also have been partly responsible for the effects. Given the type of intervention, it is not feasible to differentiate between the effects of exercise, social interaction, and departure from everyday life. Thus, the entire intervention setup might have offered QoL advantages reaching beyond the exercise factor.

## 5. Conclusions

In conclusion, this pilot study demonstrates that a one-week, ski-based exercise intervention is feasible in well-selected brain tumor patients and their relatives. As quantified by training watches, patients exercised more as compared to before and after the intervention. During the intervention, QOL measures were highest in longitudinal comparison while anxiety, depression and distress were decreased. Notably, the participating caregivers who had previously indicated high distress levels also profited from participation. These findings highlight the benefits of exercise programs in brain tumor patients and relatives while challenging general exercise discouragement in this population.

## Figures and Tables

**Table 1:** Patient characteristics.

Characteristic	Patients (*n* = 9) Median (Range)/*n*	Relatives (*n* = 6) Median (Range)/*n*
General patient characteristics		
Age (years)	47 (29–77)	49 (39–67)
Gender		
Male	5	3
Female	4	3
Weight (kg)	80 (56–100)	79 (61–90)
Height (cm)	176 (171–192)	175.5 (167–185)
BMI (kg/m^2^)	24.3 (18.9–32.3)	24.5 (20.4–28.4)
Patient diagnosis, therapy and treatment		
Diagnosis		
Multilocular meningioma grade I	1	
Oligodendroglioma grade II	1	
Astrocytoma grade II	3	
Astrocytoma grade III	1	
Glioblastoma grade IV	3	
Clinical symptoms at time of diagnosis		
Epileptic seizures	2	
Cephalgia	2	
Visual impairment	2	
Speech impairment	3	
Prior surgical therapy		
Tumor resection	9	
Re-resection	5	
Re-re-resection	1	
Prior radiotherapy		
Adjuvant radiation	7	
54 Gy w/o chemotherapy	1	
54 Gy with temozolomide	2	
59.4 Gy with temozolomide	4	
No radiation	2	
Prior chemotherapy		
Temozolomide	6	
CCNU (Lomustine)	2	
Procarbazine, CCNU & vincristine (PCV)	1	
Tumor-treating fields	1	
Photodynamic therapy *	1	
Under ongoing therapy		
Temozolomide	2	
Tumor-treating fields	1	
Social status and prior exercise		
Marital status		
Married	7	5
Single	1	0
In a relationship	1	1
Exercise frequency		
Infrequent	3	2
Sometimes	2	3
Often	4	1
Prior skiing experience		
No prior experience	2	1
Some experience, no proficiency	3	1
Some proficiency	3	4
Proficiency	1	0

* individual curative trial. BMI = body mass index; CCNU = 1-(2-chloroethyl)-3-cyclohexyl-1-nitrosourea.

## Supplementary Materials

The following are available online at https://www.mdpi.com/2077-0383/9/4/1006/s1, Figure S1: Cumulative time all participants wore their fitness watch. Figure S2: No significant changes were observed over time in two of the ASKU items, ranked from 1 (worst) to 5 (best). Figure S3: Ratio between psychological items (HADS-A, HADS-D, and Distress thermometer, respectively) and physical problem score (20 physical problem items were evaluated as present/not present). Table S1: List of adverse events used for daily checks.
