# Supplementary material for: Feasibility, Safety and Effects of a One-Week, Ski-Based Exercise Intervention in Brain Tumor Patients and Their Relatives: A Pilot Study
Source: J Clin Med. 2020 Apr 2;9(4):1006. doi: 10.3390/jcm9041006 (PMC7231125; doi:10.3390/jcm9041006)
Supplement: Supplementary file 1 [file jcm-09-01006-s001.pdf]

Supplementary Materials:

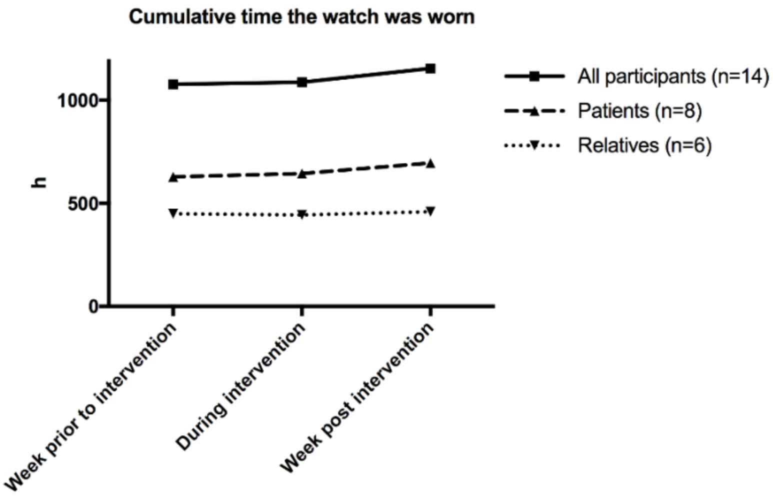

Figure S1. Cumulative time all participants wore their fitness watch. As no strong differences were observed, direct comparisons were feasible. For this figure, sums are given for all participants as well as individually for patients and relatives.

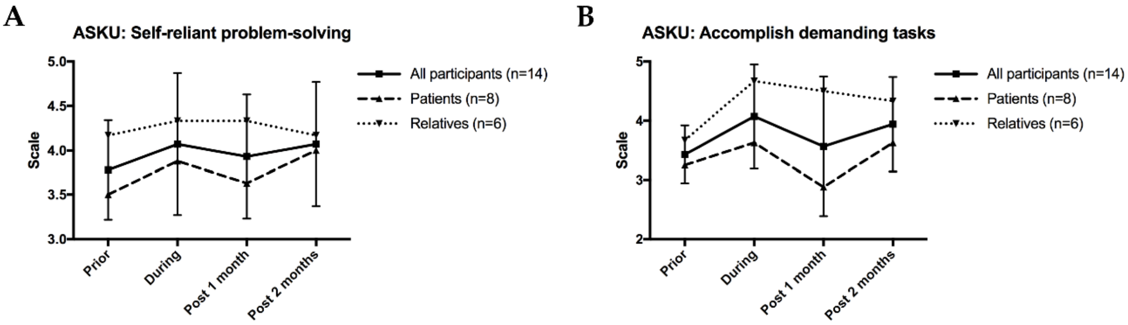

Figure S2. No significant changes were observed over time in two of the ASKU items, ranked from 1 (worst) to 5 (best). For all figures, mean results are given for all participants as well as individually for patients and relatives. The standard deviation bars were added for "All patients".

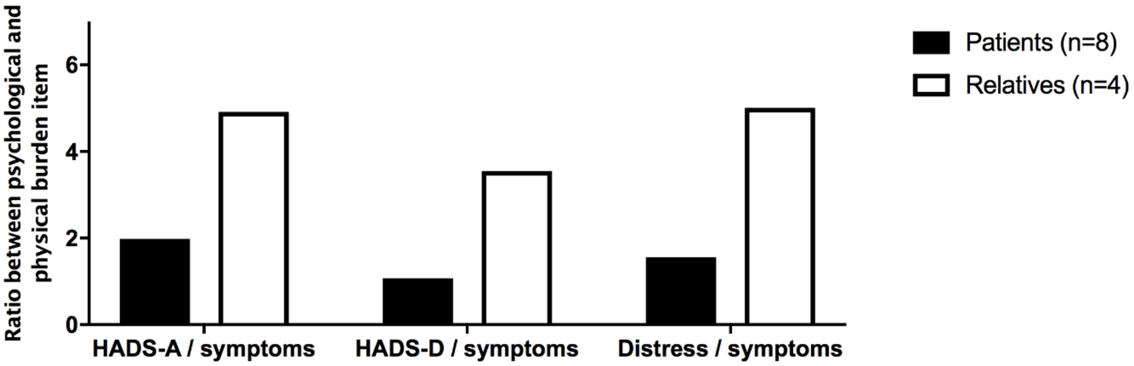

Figure S3. Ratio between psychological items (HADS-A, HADS-D, and Distress thermometer, respectively) and physical problem score (20 physical problem items were evaluated as present / not present). All participants who completed all four items across all four timepoints were included. Values were averaged across all time points and all patients or relatives.

Table 1. List of adverse events used for daily checks.

Serious Adverse Events	Non-Serious Adverse Events
• Death<br>• Reanimation / Cardiac arrest<br>• Fractures & other major traumatic injuries to the soft tissues<br>• Any other event requiring surgery / hospitalization	• Epileptic seizures not requiring hospitalization<br>• Nausea / vomiting<br>• Muscular aches<br>• Circulatory problems<br>• Superficial cuts and other wounds
